# Supplementary material for: Hybrid Models and Biological Model Reduction with PyDSTool
Source: PLoS Comput Biol. 2012 Aug 9;8(8):e1002628. doi: 10.1371/journal.pcbi.1002628 (PMC3415397; doi:10.1371/journal.pcbi.1002628)
Supplement: Text S4 — Complete source code for the PyDSTool package (version 0.88.120504). Includes API documentation and help files linking to web pages. This file is identical to the current public release on Sourceforge.net. (ZIP) [file pcbi.1002628.s004.zip › PyDSTool/html/PyDSTool.Toolbox.optimizers.criterion.criteria-module.html]

xml version="1.0" encoding="ascii"?


PyDSTool.Toolbox.optimizers.criterion.criteria


| Home | Trees | Indices | Help | PyDSTool |
| --- | --- | --- | --- | --- |

Package PyDSTool ::
Package Toolbox ::
Package optimizers ::
Package criterion ::
Module criteria

[hide private]

[frames] | no frames]

# Module criteria

source code

A list of standard convergence criteria based on the number of
iterations, the last values taken by the cost function and the associated
points


|  |  |
| --- | --- |
| Classes | [hide private] |

IterationCriterion  
A simple criterion that stops when the iteration limit is reached

MonotonyCriterion  
A simple criterion that stops when the values of the function
starts to rise again

RelativeValueCriterion  
The relative criterion stops the optimization when the relative
error of the value is below a certain level (ftol)

AbsoluteValueCriterion  
The absolute criterion stops the optimization when the absolute
error of the value is below a certain level (ftol)

RelativeParametersCriterion  
The relative criterion stops the optimization when the relative
error of the parameters is below a certain level (xtol)

AbsoluteParametersCriterion  
The absolute criterion stops the optimization when the relative
error of the parameters is below a certain level (xtol)

GradientCriterion  
The gradient criterion stops the optimization when the gradient at
the current point is less that a given tolerance

| Home | Trees | Indices | Help | PyDSTool |
| --- | --- | --- | --- | --- |

|  |  |
| --- | --- |
| Generated by Epydoc 3.0.1 on Fri May 4 15:24:05 2012 | http://epydoc.sourceforge.net |
